# Effects of garden cress, fenugreek and black seed on the pharmacodynamics of metoprolol: an herb-drug interaction study in rats with hypertension

**DOI:** 10.1080/13880209.2021.1961817

**Published:** 2021-08-15

**Authors:** Yousef A. Bin Jardan, Abdul Ahad, Mohammad Raish, Mohd Aftab Alam, Abdullah M. Al-Mohizea, Fahad I. Al-Jenoobi

**Affiliations:** Department of Pharmaceutics, College of Pharmacy, King Saud University, Riyadh, Saudi Arabia

**Keywords:** Blood pressure, CYP2D6, herbal medicine, *Lepidium sativum*, *Nigella sativa*, *Trigonella foenum-graecum*

## Abstract

**Context:**

Garden cress (GC), fenugreek (FG), and black seed (BS) are traditional herbal medicine for managing hypertension.

**Objective:**

The effects of the three herbs on the pharmacodynamics of metoprolol tartrate (MT) in hypertensive rats were investigated.

**Materials and methods:**

Wistar rats were divided in five groups (*n* = 6). Group I served as normal control group and Group II (hypertensive control group) had rats treated orally with *N*-nitro L-arginine methyl ester (L-NAME, 40 mg/kg/day) only. Groups III, IV, and V rats were orally treated with L-NAME (40 mg/kg/day) + GC (300 mg/kg, once daily), L-NAME (40 mg/kg/day) + FG (300 mg/kg, once daily) and L-NAME (40 mg/kg/day) + BS (300 mg/kg, once daily), respectively, for 2 weeks, and on the 14th day, blood pressure and heart rate were recorded using a tail-cuff blood pressure-measuring system. On the 16th day, a single dose of MT (10 mg/kg) was orally administered, and the rats’ blood pressure and heart rate were recorded.

**Results:**

GC, FG, and BS decreased systolic blood pressure (SBP) by 8.7%, 8.5%, and 8.7%, respectively, in hypertensive rats. A greater decrease in SBP by 14.5%, 14.8%, and 16.1% was observed when hypertensive rats were treated with L-NAME + GC + MT, L-NAME + FG + MT, and L-NAME + BS + MT, respectively. Similarly, hypertensive rats treated with the combination of herbs and MT had significantly lower diastolic blood pressure (DBP) than those treated with herbs alone and those treated with L-NAME alone.

**Conclusions:**

The combination of investigated herbs and MT had a beneficial effect on hypertension. However, the concurrent administration of drugs, particularly those predominantly cleared through CYP450 2D6-catalyzed metabolism, with the three investigated herbs should be considered with caution.

## Introduction

Hypertension is defined as systolic blood pressure (SBP) of more than or equal to 140 mmHg and/or diastolic blood pressure (DBP) of more than or equal to 90 mmHg (Mills et al. 2016, 2020). It is a prominent risk factor for other cardiovascular diseases and premature mortality worldwide. Approximately 31.1% of the overall population worldwide had hypertension in 2010 (Mills et al. 2016, 2020). The preponderance of hypertension is expanding, which could be due to a lack of physical activity or unhealthy diets (Mills et al. 2016). Many antihypertensive drugs are used to treat high BP levels in patients (Archer 2000; Susalit et al. 2011).

Approximately 75%–80% of the world population consume herbal medicines, mostly in developing countries, to treat diseases, including hypertension (Tabassum and Ahmad 2011). Several herbs are commonly taken by patients, especially for hypertension (Mansoor 2001; Agrawal et al. 2010; Tabassum and Ahmad 2011). These herbs might stimulate substantial herbal- medication interactions and could alter the pharmacodynamics of certain drugs and induce toxicities, especially for drugs with narrow therapeutic indices; thus, this concern should be studied more (Bushra et al. 2011; Fasinu et al. 2012; Palleria et al. 2013).

In this study, the effects of garden cress (GC), fenugreek (FG), and black seed (BS) on the pharmacodynamics of metoprolol tartrate (MT) have been determined in *N*-nitro l-arginine methyl ester (L-NAME)-induced hypertensive rats.

GC, the dried ripe seeds of *Lepidium sativum* Linn. (Cruciferae), is grown largely in Egypt and West Asia. The major constituent of GC is glucosinolates (Al-Jenoobi et al. 2015). The plant's seeds and leaves contain volatile oils and are high in fatty acids, amino acids, and minerals. Seeds are rich in carbohydrates (33–54%), protein (25%), lipids (14–24%), and crude fibre (8%). The main fatty acid in *L. sativum* seed is α-linolenic acid (32–34.0%). Stearic, linoleic, palmitic, oleic, arachidic, benzyl isothiocyanate, lignoceric acids, sitosterol and sterol are all contained in the seed oil (Prajapati et al. 2014; Chatoui et al. 2020; Ahmad et al. 2021). Other constituents include ascorbic acid, cucurbitacins, and cardenolides (Al-Jenoobi et al. 2013). An anti-hypertensive consequence of GC (20 mg/kg) has been described in spontaneously hypertensive rats but not in Wistar Kyoto rats (normotensive control). It was reported that the increase in urinary removal of chlorides, sodium and potassium could be the cause for its blood pressure lowering effect (Maghrani et al. 2005).

GC has also been used for managing respiratory disorders, vitamin C deficiency, constipation, and poor immunity and as a diuretic (Al-Jenoobi et al. 2013). Practitioners of Indian medicine consider its seeds useful in managing dysenteric diarrhoea and febrile and catarrhal infections (Rehman et al. 2012). Moreover, GC is widely consumed as a food-nutrient ingredient for cooking and freshly prepared salads. In addition, GC is rich in dietary fibre and contains more than 20% protein (Doke and Guha 2014).

FG [*Trigonella foenum-graecum* Linn (Fabaceae)] is indigenous to Eastern Europe and parts of Asia; however, it has now been extensively cultivated in nearly all countries. Its leaves and seeds are usually used as leafy vegetables and condiments, respectively (Reddy and Srinivasan 2011; Yadav and Baquer 2014). A mature FG seed has many active components, such as fenugreekine, diosgenin, gitogenin, neogitogenin, homoorientin, saponaretin, neogigogenin, tigogenin, fibres, flavonoids, polysaccharides, fixed oils, and some identified alkaloids, that is, carpaine, gentianine, trigonelline and choline (Yoshikawa et al. 1997; Morani et al. 2012; Nagulapalli Venkata et al. 2017). It was reported that the serotonergic antagonistic property of the 5-HT_2_ subtype receptor could play a role in substantial decrease in high blood pressure in rats (Balaraman et al. 2006). FG seeds have several activities, including antihypertensive, antiperoxidative, hypocholesterolemic, and antidiabetic effects and anti-inflammatory and insulin-mimetic properties (Madar and Shomer 1990; Petit et al. 1995; Nair et al. 1998; Patel et al. 2012; Geberemeskel et al. 2019).

BS [*Nigella sativa* Linn (Ranunculaceae)] is an annual herb with enormous therapeutic potential (Kooti et al. 2016). The beneficial potential of this plant is primarily attributed to the free radical scavenging properties of some of its active components (Darakhshan et al. 2015; Gholamnezhad et al. 2016). BS seed oils comprise two active components, thymoquinone and dihydrothymoquinone, which demonstrated strong antioxidant potential (Darakhshan et al. 2015; Butt et al. 2019). Other active constituents have been found in BS are 4-terpineol, α-pinene, carvacrol, limonene, longifolene, p-cymene, t-anethole benzene and thymol (Kooti et al. 2016; Ijaz et al. 2017; Tavakkoli et al. 2017). The actual mechanism by which BS decreases the blood pressure is unclear. The many active compounds in BS may be responsible for its antihypertensive effects, each with its own mechanism of action. The antioxidant, diuretic, calcium channel blocking property and cardiac depressant effect are potential mechanisms they may help to lower blood pressure (Dehkordi and Kamkhah 2008; Jaarin et al. 2015; Rizka et al. 2017). BS has great pharmacological potency in managing various diseases, including cardiovascular diseases, diabetes, and hypertension (Jaarin et al. 2015; Rizka et al. 2017; Enayatfard et al. 2018; Xiao et al. 2018; Hamdan et al. 2019). In addition, it demonstrated chemo-protective, gastro-protective, and immune-protective activities (Amin and Hosseinzadeh 2016; Ijaz et al. 2017; Majdalawieh et al. 2017; Mollazadeh et al. 2017).

MT is commonly used in managing acute myocardial infarction, angina, hypertension, and cardiac arrhythmias (Ripley and Saseen 2014; Ahad et al. 2015; Grassi 2018). It belongs to the therapeutic group of selective β-adrenergic blockers. The enteral absorption of MT is quick and nearly complete; nevertheless, the bioavailability of MT is 50%, which could be due to the broad first-pass metabolism (Berger et al. 2018). MT is comprehensively bio-transformed, with only less than 5% of an oral dose being excreted in a non-metabolised form by the kidneys (Regardh and Johnsson 1980; Johansson et al. 2007). Almost 70% of orally taken MT is mainly metabolised by cytochrome P450 2D6 (CYP2D6) (Berger et al. 2018). The half-life of MT is in the ranges of 3–4 h and 7–9 h in young adults and elderly patients, respectively (Rigby et al. 1985).

Since these herbs (GC, FG, and BS) have demonstrated antihypertensive activity, consuming these herbs has a reasonable potential for lowering high blood pressure levels, alone or with another antihypertensive herb or modern medicines. Hence, exploring the effects of these herbs on the pharmacodynamics of MT is essential. This study evaluated the influence of these herbs on the pharmacodynamics of MT in diseased rats.

## Materials and methods

### Materials

Lopressor^®^ 50 (MT) was purchased from Novartis Pharma AG, Basle, Switzerland. L-NAME was purchased from Carbosynth Limited, Berkshire, UK. GC, FG, and BS were procured from the production of 7 Spices Trading Establishment, Riyadh, Saudi Arabia. The herbs were previously authenticated by expert taxonomist of the university and all investigated herbs were procured from the same source.

### Animals

Wistar rats with weights between 200 and 250 g were received after the study was approved by the Research Ethics Committee. The rats were divided into five groups having 6 rats in each group. All animal management and handling procedures, in addition to the study design, were approved by the Ethics Committee of King Saud University (KSU-SE-18-27).

### Induction of hypertension

Oral administration of L-NAME at a dose of 40 mg/kg/day was used for inducing hypertension in each group of rats (except for Group I). Rats showing SBP of more than 150 mmHg were included in this study (Adaramoye et al. 2012; Sung et al. 2013; Ahad et al. 2020a, 2020b).

### Training of rats and recording of blood pressure

Tail-cuff systems are usually employed for observing blood pressure in rats and mice (Krege et al. 1995; Feng et al. 2009; Ahad et al. 2014). Training of rats for the tail-cuff measurement of blood pressure is an important step because factors, such as warming of rat platforms and sitting of rats in a restrainer that involved in the procedure could affect the blood pressure of the animals (Zhao et al. 2011; Ahad et al. 2015, 2017, 2018). Primarily, gripping the animals gently is essential so that they remained in a state of proper calmness. To lessen the consequences of this variability, the rats were trained for 10–15 min/day in the restrainer for 5 days. The rat restrainer has a proper ventilation window, and the other side of the restrainer has an opening by which the tail is protruded outside and passes via the tail cuff and V-shaped groove in the sensor. The restrainer was placed above the platform, which was lightly heated using a temperature controller to gently warm the rats so that blood flow to the tail was established, which in-turn produced good signals from the rat tail that were recognised by the sensor. The signals were read by the control unit during automatic inflation/deflation of the tail cuff, and these signals were interpreted by the control unit, which displayed the blood pressure curve in the attached computer. During experiments, decrements in the heart rate (HR) of the rats with training were observed, and this decrease in the HR of rats could be most probably because of a decrease in animal stress. A substantial decrease in the HR of the trained rats was observed compared with that of untrained rats.

### Experimental design

The animals were divided into five groups (*n* = 6): Group I served as the healthy control group. Hypertension was induced in the animals of Groups II-V. Group II was labelled as the hypertensive control group where the rats were orally treated with L-NAME only (40 mg/kg, once daily). Groups III, IV, and V animals were orally treated with L-NAME + GC (300 mg/kg, once daily), L-NAME + FG (300 mg/kg, once daily), and L-NAME + BS (300 mg/kg, once daily), respectively. The treatment of Groups II, III, IV, and V animals lasted for two weeks, and on the 14th day, the SBP, DBP, HR, and mean arterial pressure (MAP) of each rat of every group were recorded at 0, 1, 2, 4, 8, 12, and 24 h using a tail-cuff blood pressure measuring system (BP-2000 Blood Pressure Analysis System; Visitech Systems, NC, USA). On the 16th day, a single dose of MT (10 mg/kg, orally) was administered to each rat, and SBP, DBP, HR, and MAP of every rat of Groups II-V were recorded at 0, 1, 2, 4, 8, 12, and 24 h.

### Statistical analysis

Differences in the means were analysed using one-way ANOVA, followed by Dunnett’s multiple comparisons test using Prism 6.00 (GraphPad Software, Inc, CA, USA). *p-values of less than 0.05 and **p-values of less than 0.01 were considered statistically significant.

## Results and discussion

The SBP, DBP, MAP, and HR were monitored in all groups using a tail-cuff BP-2000 Blood Pressure Analysis System (Visitech Systems, NC, USA). The antihypertensive effect of the three herbs, namely, GC, FG, and BS, were compared in contrast to the hypertensive control group. The healthy control group showed SBP, DBP, and MAP in the ranges of 110–126 mmHg, 62–84 mmHg, and 81–95 mmHg, respectively. The L-NAME model emulated hypertension in humans, and it is a well-proven experimental model for inducing hypertension in animals (Ramanathan and Thekkumalai 2014). In this study, hypertension was successfully induced with L-NAME, and rats were screened as hypertensive rats and used for the experiment. In this study, a substantial increase in SBP, DBP, and MAP was correlated with the oral administration of L-NAME compared with those in the healthy control rats, corroborating the induction of hypertension in Group II-V animals. Rats treated with L-NAME (40 mg/kg/day) had higher SBP, DBP, and MAP by 45%, 37%, and 40%, respectively, than healthy control rats. Our results conform to those of other published studies where L-NAME induced a persistent increase in blood pressure (Cardoso et al. 2014; Jaarin et al. 2015; Abdel-Rahman et al. 2017).

### Effects of herbs on SBP in hypertensive rats

**Figure 1.**
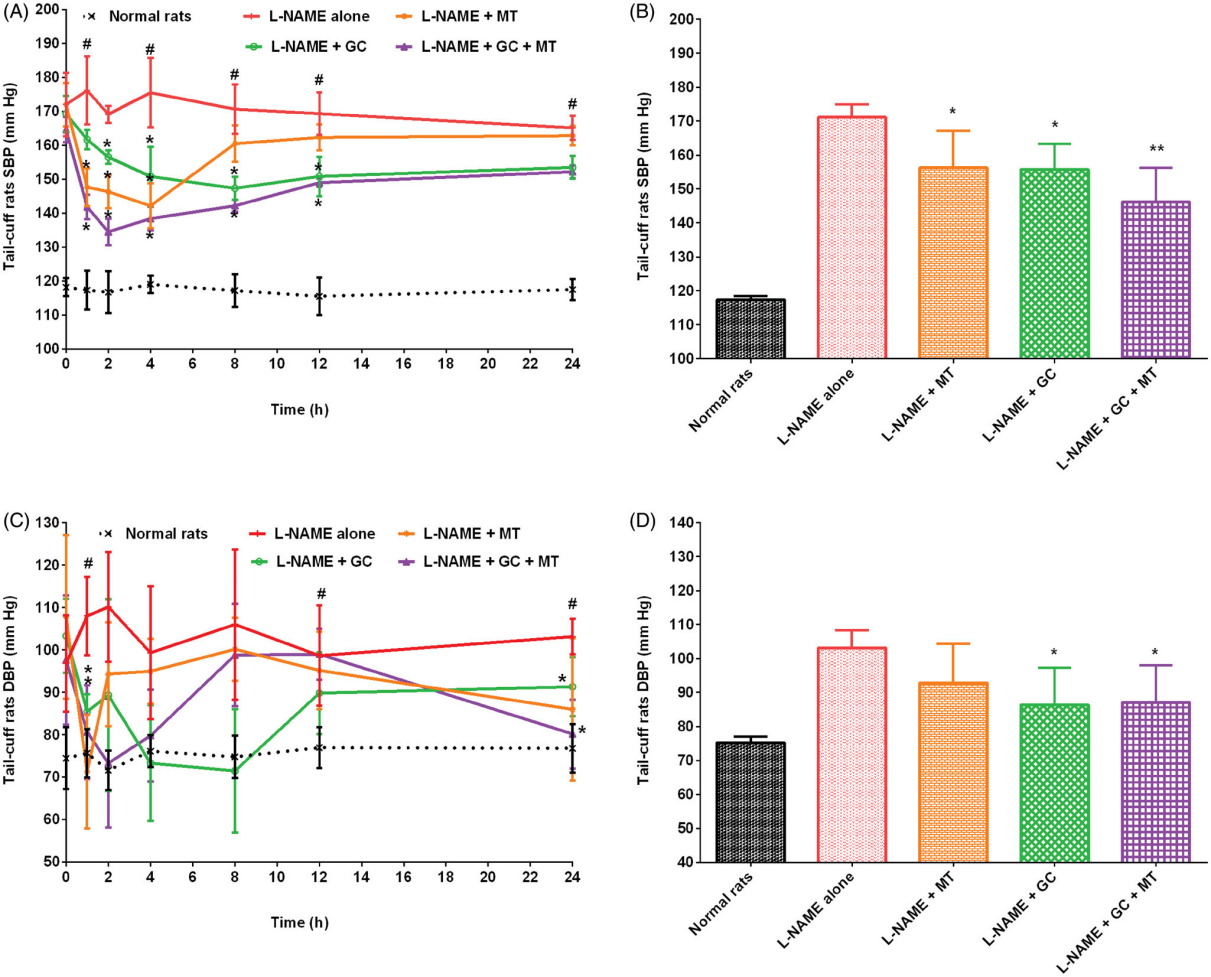
Effects of garden cress with and without MT on SBP and DBP of hypertensive rats (mean ± SD). (A and C) Time course effects (B and D) mean 0–24 h time point. *p < 0.05, **p < 0.01 versus L-NAME alone; **^#^***p* < 0.01 versus normal rats. DBP: diastolic blood pressure; MT: metoprolol tartrate; SBP: systolic blood pressure.

**Figure 2.**
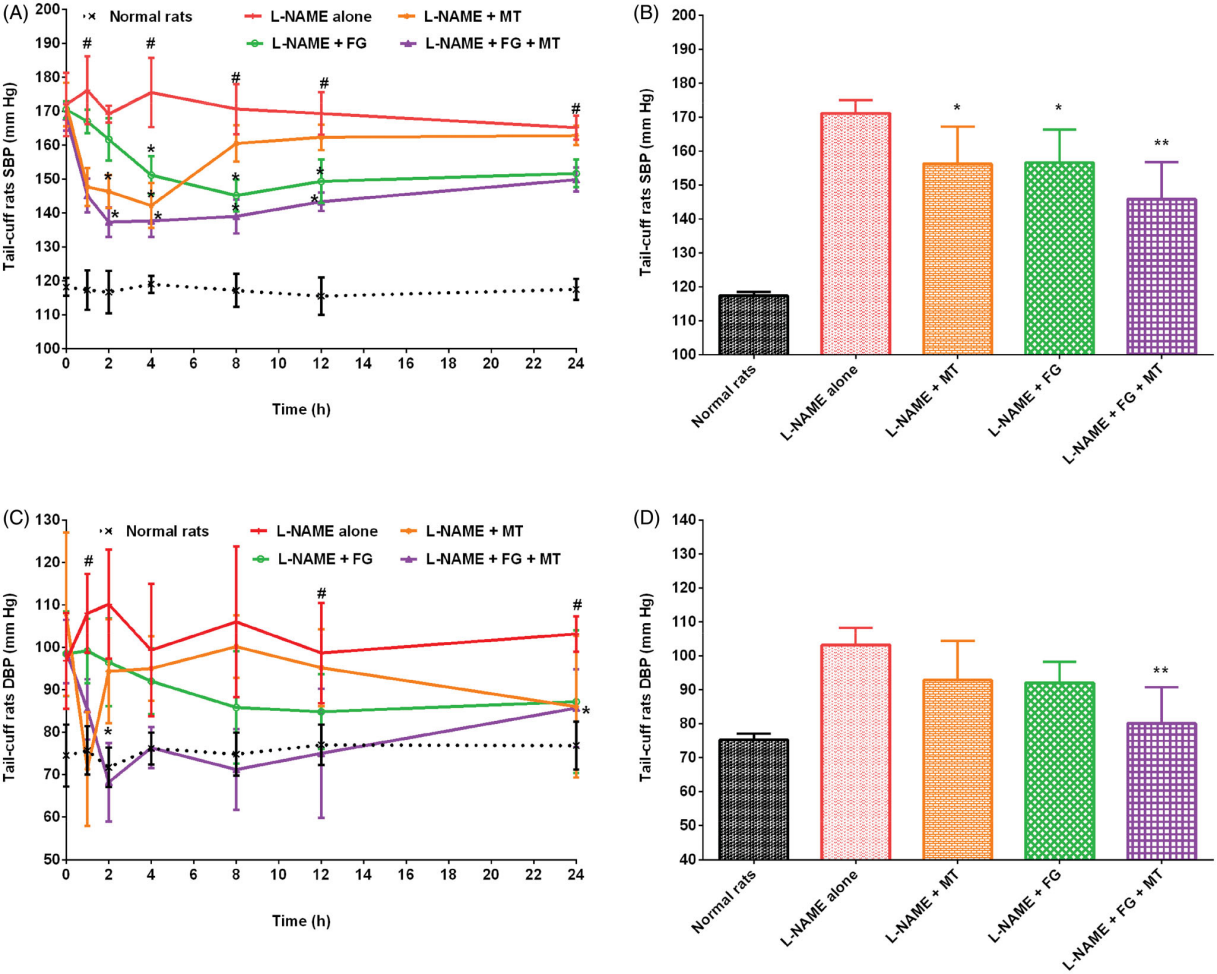
Effects of fenugreek with and without MT on SBP and DBP of hypertensive rats (mean ± SD). (A and C) Time course effects (B and D) mean 0–24 h time point. *p < 0.05, **p < 0.01 versus L-NAME alone; **^#^***p* < 0.01 versus normal rats. DBP: diastolic blood pressure; MT: metoprolol tartrate; SBP: systolic blood pressure.

**Figure 3.**
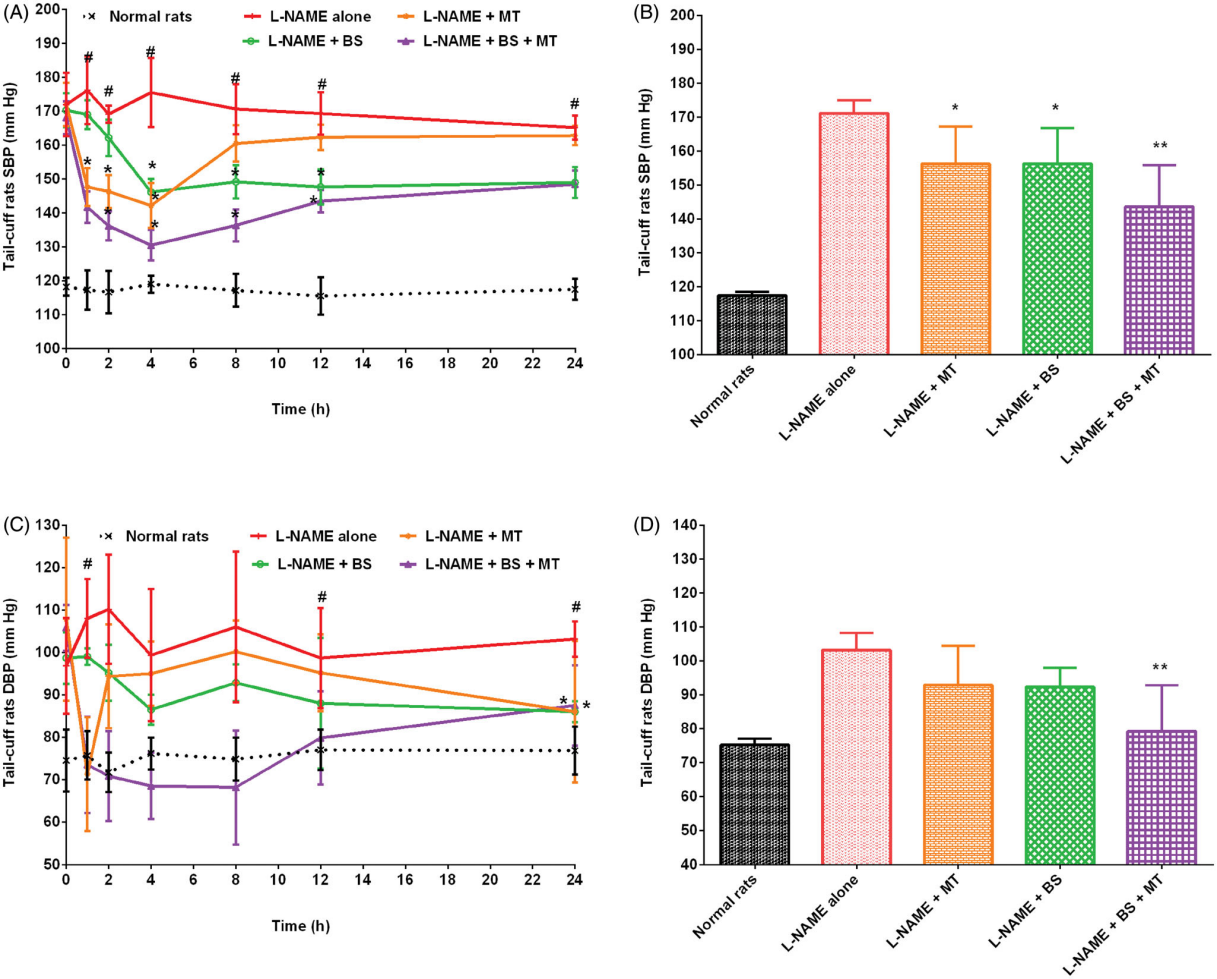
Effects of black seed with and without MT on SBP and DBP of hypertensive rats (mean ± SD). (A and C) Time course effects (B and D) mean 0–24 h time point. *p < 0.05, **p < 0.01 versus L-NAME alone; **^#^***p* < 0.01 versus normal rats. DBP: diastolic blood pressure; MT: metoprolol tartrate; SBP: systolic blood pressure.

The healthy control and hypertensive control rats (treated with L-NAME alone) groups showed SBP in the ranges of 110–126 mmHg and 159–193 mmHg (*p* < 0.01), respectively. The administration of MT decreased SBP in hypertensive rats. Rats treated with L-NAME + MT had a significant (*p* < 0.05) decrease in SBP by 8.7% compared with the hypertensive control group. The maximum decrease in SBP (19%; 142.17 ± 6.65 mmHg, *p* < 0.05) was observed 4 h after MT administration. SBP gradually increased after 4 h and reached 162.83 ± 2.86 mmHg after 24 h of MT treatment. GC caused a significant (*p* < 0.05) drop in SBP in hypertensive rats (Figure 1). In addition, GC reduced SBP in hypertensive rats by 9% compared with that in the hypertensive control group (treated with L-NAME alone). The maximum antihypertensive effect of L-NAME + GC treatment was observed after 8 h; the SBP decreased by 13% (*p* < 0.05) at 8 h compared with that in the hypertensive control group (treated with L-NAME alone). Meanwhile, rats treated with L-NAME + GC + MT had decreased SBP by 14.5%, 6.5%, and 6.1% compared with rats treated with L-NAME alone, L-NAME + MT, and L-NAME + GC, respectively (Figure 1). The maximum decrease in SBP (20%; 134.50 ± 3.89 mmHg, *p* < 0.05) was observed 2 h after L-NAME + GC + MT treatment compared with that in rats treated with L-NAME alone. Similarly, oral treatment of hypertensive rats with FG at a dose of 300 mg/kg/day showed a significant (*p* < 0.05) drop in SBP (Figure 2). Rats treated with L-NAME + FG showed a decrease in SBP by 8.5% compared with animals treated with L-NAME alone. A maximum drop in SBP (15%; 145.17 ± 4.67 mmHg, *p* < 0.05) was noted 8 h after treatment. The hypotensive effect of FG began to decrease after 8 h, and SBP rose to 151.67 ± 4.03 mmHg at 24 h. L-NAME + FG + MT showed a significant (*p* < 0.01) decrease in SBP by 14.8% compared with those in rats treated with L-NAME alone. At 2 h, an 18.8% (*p* < 0.01) decrease in SBP was noted compared with those in rats treated with L-NAME alone. The treatment of rats with L-NAME + FG + MT decreased SBP by 6.7% and 6.9% compared with treatment with L-NAME + MT and L-NAME + FG, respectively (Figure 2). Alternatively, the treatment of hypertensive rats with BS decreased the SBP by 8.7% after 24 h compared with treatment with L-NAME alone (Figure 3). The maximum decrease in SBP (16.7%; 146.17 ± 3.76 mmHg, *p* < 0.05) was observed 4 h after BS administration. The blood pressure lowering effect of BS started to diminish after 4 h, and SBP began to rise gradually and reached 149.00 ± 4.60 mmHg. Animals treated with L-NAME + BS + MT showed a decrease in SBP by 16.1% (*p* < 0.05) compared with the rats in the hypertensive control group. The maximum decrease in SBP (25.6%; 130.50 ± 4.51 mmHg, *p* < 0.05) was observed 4 h after L-NAME + BS + MT treatment. The treatment of rats with L-NAME + BS + MT decreased SBP by 16.1%, 8.1%, and 8.1% compared with treatment with L-NAME alone, L-NAME + MT, and L-NAME + BS, respectively (Figure 3).

### Effects of herbs on DBP in hypertensive rats

In this study, the DBP of rats were in the ranges of 62–86 mmHg and 77–127 mmHg in the healthy control and hypertensive control groups. In this study, all investigated herbs substantially reduced the DBP of hypertensive rats. Rats treated with L-NAME + MT demonstrated a decrease in DBP by 10% compared with those treated with L-NAME alone. The administration of MT resulted in a sudden decrease in DBP in rats with L-NAME-induced hypertension, with a maximum decrease of 34% (71.33 ± 13.44 mmHg) at 1 h. The results indicated that DBP considerably decreased in rats treated with L-NAME + GC compared with that in hypertensive control rats (Figure 1). Animals treated with GC showed a maximum decrease in DBP of 32% (71.50 ± 14.52 mmHg, *p* < 0.01) at 8 h compared with the hypertensive control group. A 33.4% (*p* < 0.01) reduction in DBP was observed at an earlier time point of 2 h when rats were treated with L-NAME + GC + MT (Figure 1). Animals treated with L-NAME + GC + MT presented with a substantial decrease in DBP by 15.61%, 6.21%, and 0.88% compared with rats treated with L-NAME alone, L-NAME + MT, and L-NAME + GC, respectively. The treatment of hypertensive rats with L-NAME + FG and L-NAME + BS decreased DBP by 10.82% and 10.52%, respectively, after 24 h compared with treatment with L-NAME alone (Figures 2 and 3). Animals treated with L-NAME + FG + MT and L-NAME + BS + MT showed a significant (*p* < 0.05) decrease in DBP by 22.36% and 23.26%, respectively, compared with the hypertensive control group (Figures 2 and 3). The maximum decreases in DBP of 38.12% (68.17 ± 9.28 mmHg, *p* < 0.05) and 31.04% (68.50 ± 7.82 mmHg) were noted 2 h and 4 h, respectively, after L-NAME + FG + MT and L-NAME + BS + MT treatment. The treatment of rats with L-NAME + FG + MT decreased DBP by 13.72% and 12.94% compared with treatment with L-NAME + MT and L-NAME + FG, respectively. Meanwhile, animals treated with L-NAME + BS + MT had a decrease in DBP by 14.72% and 14.24% compared with those treated with L-NAME + MT and L-NAME + BS, respectively.

### Effects of herbs on MAP in hypertensive rats

**Figure 4.**
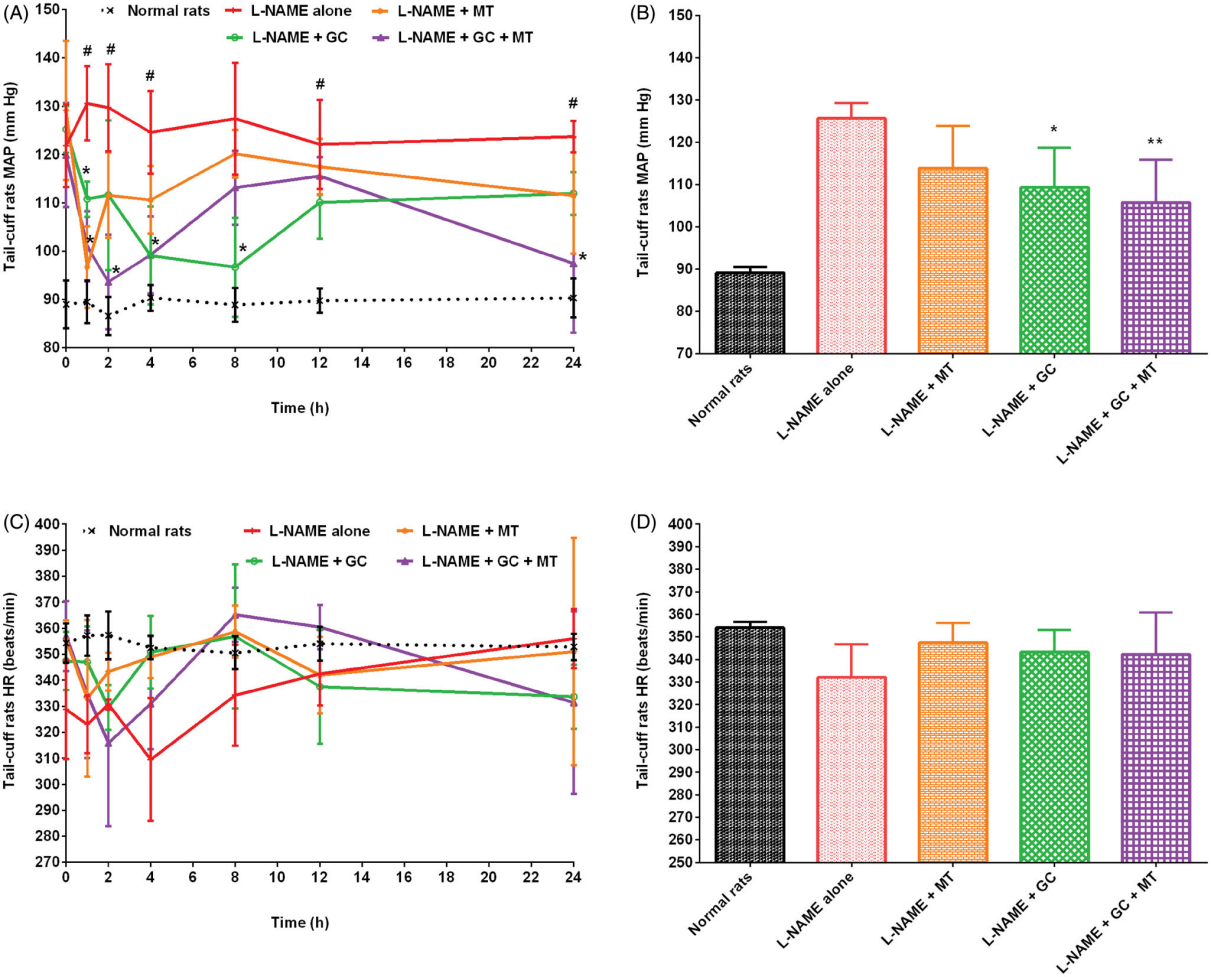
Effects of garden cress with and without MT on MAP and HR of hypertensive rats (mean ± SD). (A and C) Time course effects (B and D) mean 0–24 h time point. *p < 0.05, **p < 0.01 versus L-NAME alone; **^#^***p* < 0.01 versus normal rats. HR: heart rate; MAP: mean arterial pressure; MT: metoprolol tartrate.

**Figure 5.**
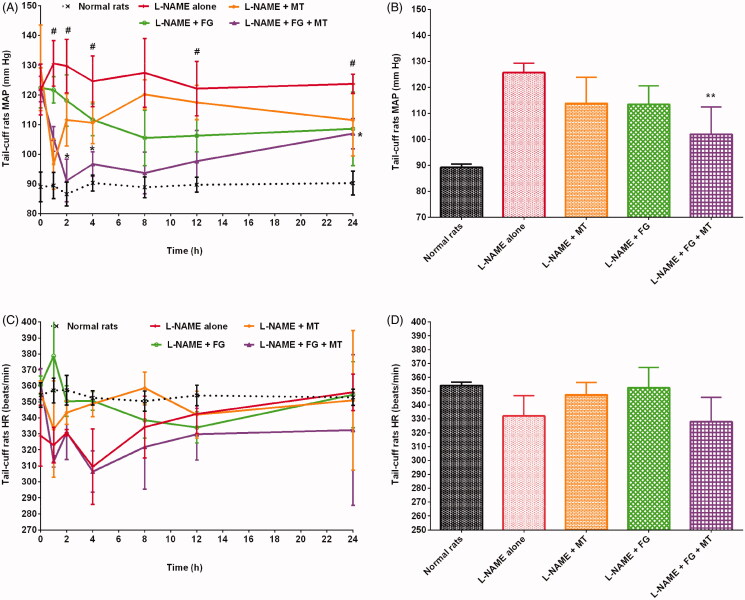
Effects of fenugreek with and without MT on MAP and HR of hypertensive rats (mean ± SD). (A and C) Time course effects (B and D) mean 0–24 h time point. **p < 0.01 versus L-NAME alone; **^#^***p* < 0.01 versus normal rats. HR: heart rate; MAP: mean arterial pressure; MT: metoprolol tartrate.

**Figure 6.**
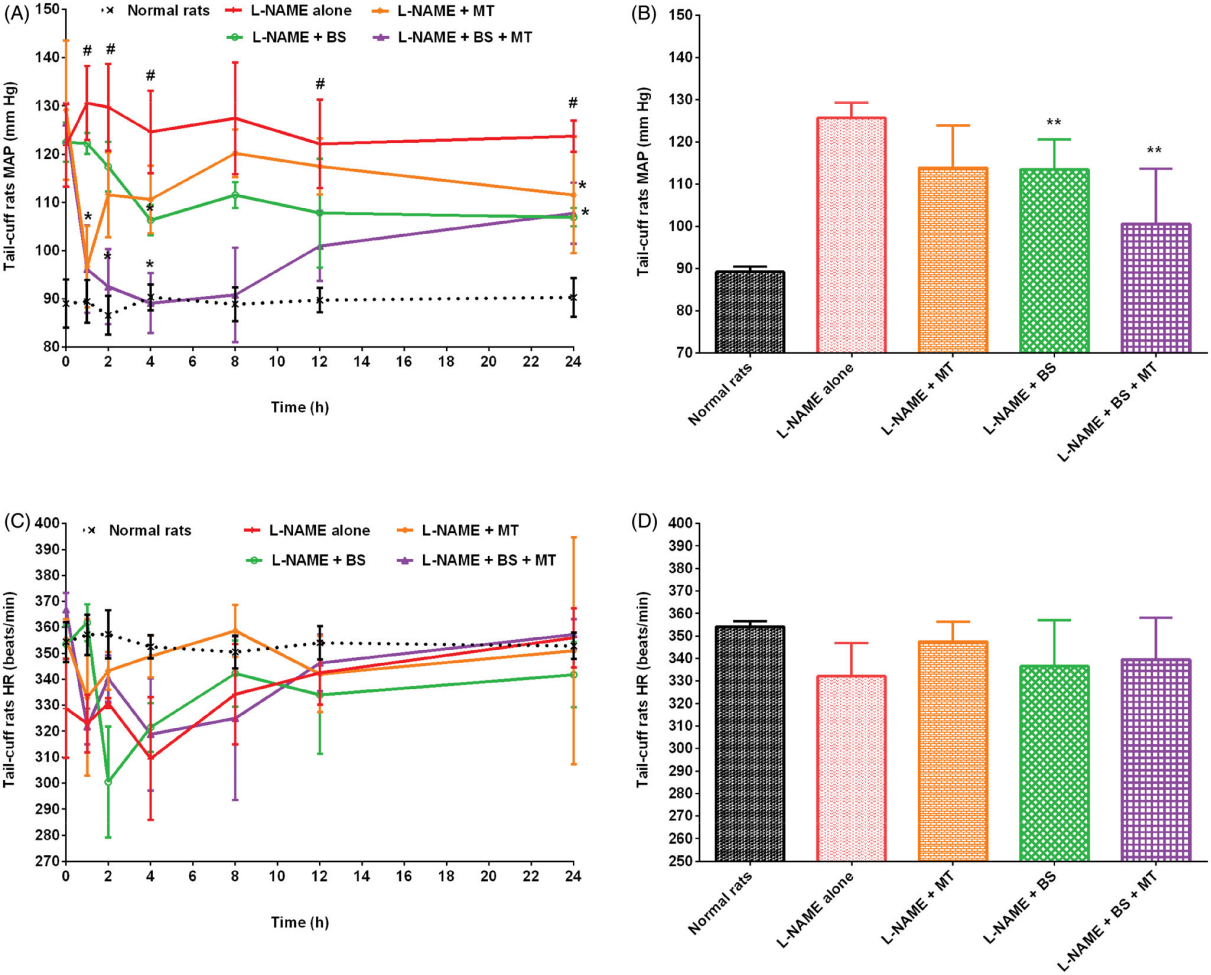
Effects of black seed with and without MT on MAP and HR of hypertensive rats (mean ± SD). (A and C) Time course effects (B and D) mean 0–24 h time point. **p < 0.01 versus L-NAME alone; **^#^***p* < 0.01 versus normal rats. HR: heart rate; MAP: mean arterial pressure; MT: metoprolol tartrate.

In this study, the MAP of rats was in the ranges of 81–95 mmHg and 107–145 mmHg in the healthy control and hypertensive control groups, respectively. Rats treated with L-NAME + MT, L-NAME + GC, L-NAME + FG, and L-NAME + BS presented a reduction in MAP by 9.42%, 13.02%, 9.76%, and 9.71%, respectively, compared with those treated with L-NAME alone (Figures 4–6). Animals treated with L-NAME + GC + MT, L-NAME + FG + MT, and L-NAME + BS + MT presented a decrease in MAP by 15.92%, 18.94%, and 20.03%, respectively, compared with rats treated with L-NAME alone (Figures 4–6). Meanwhile, rats treated with L-NAME + GC + MT, L-NAME + FG + MT, and L-NAME + BS + MT exhibited a decrease in MAP by 7.18%, 10.51%, and 11.71%, respectively, compared with those treated with L-NAME + MT. Animals that received L-NAME + GC + MT, L-NAME + FG + MT, and L-NAME + BS + MT exhibited a decrease in MAP by 3.33%, 10.17%, and 11.43%, respectively, compared with those treated with L-NAME + GC, L-NAME + FG, and L-NAME + BS.

### Effects of herbs on HR in hypertensive rats

The healthy control and hypertensive control groups showed HR in the ranges of 344–369 beats/min and 275–365 beats/min, respectively. Rats that received L-NAME alone presented a decrease in HR by 6.21% compared with healthy control rats. Our results conform to those of other studies where L-NAME administration decreased HR (Bernatova et al. 1999; Kobayashi et al. 2000; Sung et al. 2013; Abdel-Rahman et al. 2017). Animals treated with L-NAME + MT, L-NAME + GC, L-NAME + FG, and L-NAME + BS exhibited improvements in HR by 4.62%, 3.35%, 6.13%, and 1.32%, respectively, compared with those treated with L-NAME alone (Figures 4–6). Animals that received L-NAME + GC + MT and L-NAME + BS + MT had an increase in HR by 3.04% and 2.21%, respectively, compared with those treated with L-NAME alone (Figures 4–6). However, rats treated with L-NAME + FG + MT unexpectedly did not show any improvement in HR (Figure 5).

Nowadays, herbal medicines are extensively used; this could be due to their low costs and fewer side effects associated with them (Modak et al. 2007). Several studies have explored the therapeutic potentials ​​of plants to ascertain their application in current therapies. The search for commonly used herbs that mitigate hypertension brought GC, FG, and BS into light for experimentation. These three herbs were used to cure different illnesses in ancient medicine. In this study, these herbs were evaluated for their blood pressure-lowering action in rats. Furthermore, the pharmacodynamic interaction of the aforementioned herbs and MT was examined in this study.

Several studies have indicated that the administration of L-NAME over a longer period increased blood pressure (Mali et al. 2012; Bernatova 2014; Syed et al. 2016; Tata et al. 2019). In this study, we found that L-NAME increased SBP, DBP, and MAP in rats, and the subsequent hypertension was relieved by oral administration of the investigated herbs, namely, GC, FG and BS; these outcomes presented the antihypertensive effects of these herbs in animal models. In this study, test animals from the hypertensive groups, such as rats treated with L-NAME + GC, L-NAME + FG, and L-NAME + BS, showed lower SBP, DBP, and MAP than rats treated with L-NAME alone (hypertensive control). These herbs improved the HR of hypertensive rats. All three investigated herbs were more effective in reducing blood pressure in hypertensive rats. Among them, GC was slightly more effective, whereas FG and BS were comparably potent in decreasing SBP in hypertensive rats. In addition, this study showed that long-term treatment with the three herbs under study has a substantial influence in reversing hypertension. Nevertheless, this study has limitations; consequently, additional research is required to determine the active constituents of these herbs that play an important role in the molecular mechanism behind their antihypertensive action.

## Conclusions

In this study, we used L-NAME-induced hypertension models to investigate the antihypertensive effects of three commonly used herbs, namely, garden cress (GC), fenugreek (FG), and black seed (BS); for this purpose, L-NAME was orally administered in rats, which induced a substantial increase in blood pressure. The outcomes of this study showed that metoprolol tartrate (MT) alone, herbs alone, and their combination showed a decrease in systolic blood pressure (SBP), diastolic blood pressure (DBP), and mean arterial pressure (MAP) in hypertensive rats. Heart rate (HR), which was depleted following treatment with L-NAME alone, improved following treatment with MT alone, herbs alone, and herbs + MT. A more potent blood pressure-lowering effect of MT was observed when administered in combination with herbs. Furthermore, the concurrent administration of drugs, particularly those predominantly cleared through CYP2D-catalyzed metabolism, with the three herbs under study should be considered with caution.

## References

[CIT0001] Abdel-Rahman RF, Hessin AF, Abdelbaset M, Ogaly HA, Abd-Elsalam RM, Hassan SM. 2017. Antihypertensive effects of Roselle-Olive combination in L-NAME-induced hypertensive rats. Oxid Med Cell Longev. 2017:9460653. 29201276 10.1155/2017/9460653 PMC5671754

[CIT0002] Adaramoye OA, Nwosu IO, Farombi EO. 2012. Sub-acute effect of N(G)-nitro-l-arginine methyl-ester (L-NAME) on biochemical indices in rats: protective effects of kolaviron and extract of *Curcuma longa* L. Phcog Res. 4(3):127–133. 22923949 10.4103/0974-8490.99071 PMC3424838

[CIT0003] Agrawal M, Nandini D, Sharma V, Chauhan NS. 2010. Herbal remedies for treatment of hypertension. Int J Pharm Sci Res. 1:1–21.

[CIT0004] Ahad A, Al-Jenoobi FI, Al-Mohizea AM, Akhtar N, Raish M, Aqil M. 2015. Systemic delivery of β-blockers via transdermal route for hypertension . Saudi Pharm J. 23(6):587–602. 26702253 10.1016/j.jsps.2013.12.019 PMC4669430

[CIT0005] Ahad A, Al-Saleh AA, Al-Mohizea AM, Al-Jenoobi FI, Raish M, Yassin AEB, Alam MA. 2017. Pharmacodynamic study of eprosartan mesylate-loaded transfersomes Carbopol^®^ gel under Dermaroller^®^ on rats with methyl prednisolone acetate-induced hypertension. Biomed Pharmacother. 89:177–184. 28237913 10.1016/j.biopha.2017.01.164

[CIT0006] Ahad A, Aqil M, Ali A. 2014. Investigation of antihypertensive activity of carbopol valsartan transdermal gel containing 1,8-cineole. Int J Biol Macromol. 64:144–149. 24296403 10.1016/j.ijbiomac.2013.11.018

[CIT0007] Ahad A, Aqil M, Kohli K, Sultana Y, Mujeeb M. 2015. Nano vesicular lipid carriers of angiotensin II receptor blocker: anti-hypertensive and skin toxicity study in focus. Artif Cells Nanomed Biotechnol. 44:1002–1007. 25707444 10.3109/21691401.2015.1008509

[CIT0008] Ahad A, Raish M, Al-Jenoobi FI, Al-Mohizea AM. 2018. Sorbitane monostearate and cholesterol based niosomes for oral delivery of telmisartan. Curr Drug Deliv. 15(2):260–266. 28521674 10.2174/1567201814666170518131934

[CIT0009] Ahad A, Raish M, Bin Jardan YA, Alam MA, Al-Mohizea AM, Al-Jenoobi FI. 2020a. Effect of *Hibiscus sabdariffa* and *Zingiber officinale* on the antihypertensive activity and pharmacokinetic of losartan in hypertensive rats. Xenobiotica. 50(7):847–857. 32048541 10.1080/00498254.2020.1729446

[CIT0010] Ahad A, Raish M, Bin Jardan YA, Alam MA, Al-Mohizea AM, Al-Jenoobi FI. 2020b. Potential pharmacodynamic and pharmacokinetic interactions of *Nigella sativa* and *Trigonella foenum-graecum* with losartan in L-NAME induced hypertensive rats. Saudi J Biol Sci. 27(10):2544–2550. 32994710 10.1016/j.sjbs.2020.05.009 PMC7499079

[CIT0011] Ahmad A, Nabi R, Mishra A, Ahmad IZ. 2021. A panoramic review on *Lepidium sativum* L. bioactives as prospective therapeutics. Drug Res. 71(5):233–242. 10.1055/a-1334-4101 33378774

[CIT0012] Al-Jenoobi FI, Ahad A, Mahrous GM, Al-Mohizea AM, AlKharfy KM, Al-Suwayeh SA. 2015. Effects of fenugreek, garden cress, and black seed on theophylline pharmacokinetics in beagle dogs. Pharm Biol. 53(2):296–300. 25243874 10.3109/13880209.2014.916312

[CIT0013] Al-Jenoobi FI, Al-Suwayeh SA, Muzaffar I, Alam MA, Al-Kharfy KM, Korashy HM, Al-Mohizea AM, Ahad A, Raish M. 2013. Effects of *Nigella sativa* and *Lepidium sativum* on cyclosporine pharmacokinetics. Biomed Res Int. 2013:953520. 23957013 10.1155/2013/953520 PMC3730136

[CIT0014] Amin B, Hosseinzadeh H. 2016. Black cumin (*Nigella sativa*) and its active constituent, thymoquinone: an overview on the analgesic and anti-inflammatory effects. Planta Med. 82(1–2):8–16. 26366755 10.1055/s-0035-1557838

[CIT0015] Archer JS. 2000. Evaluation and treatment of hypertension. Prim. Care Update Ob Gyns. 7(1):1–6.

[CIT0016] Balaraman R, Dangwal S, Mohan M. 2006. Antihypertensive effect of *Trigonella foenumgreacum*. seeds in experimentally induced hypertension in rats. Pharm Biol. 44(8):568–575.

[CIT0017] Berger B, Bachmann F, Duthaler U, Krahenbuhl S, Haschke M. 2018. Cytochrome P450 enzymes involved in metoprolol metabolism and use of metoprolol as a CYP2D6 phenotyping probe drug. Front Pharmacol. 9:774. 30087611 10.3389/fphar.2018.00774 PMC6066528

[CIT0018] Bernatova I. 2014. Endothelial dysfunction in experimental models of arterial hypertension: cause or consequence? Biomed Res Int. 2014:598271. 24738065 10.1155/2014/598271 PMC3971506

[CIT0019] Bernatova I, Pechanova O, Simko F. 1999. Effect of captopril in L-NAME-induced hypertension on the rat myocardium, aorta, brain and kidney. Exp Physiol. 84(6):1095–1105. 10564706

[CIT0020] Bushra R, Aslam N, Khan AY. 2011. Food-drug interactions. Oman Med J. 26(2):77–83. 22043389 10.5001/omj.2011.21 PMC3191675

[CIT0021] Butt AS, Nisar N, Mughal TA, Ghani N, Altaf I. 2019. Anti-oxidative and anti-proliferative activities of extracted phytochemical compound thymoquinone. J Pak Med Assoc. 69(0):1–1485. 31622301

[CIT0022] Cardoso AM, Abdalla FH, Bagatini MD, Martins CC, da Silva Fiorin F, Baldissarelli J, Costa P, de Mello FF, Fiorenza AM, da Silva Serres JD, et al. 2014. Swimming training prevents alterations in acetylcholinesterase and butyrylcholinesterase activities in hypertensive rats. Am J Hypertens. 27(4):522–529. 23479073 10.1093/ajh/hpt030

[CIT0023] Chatoui K, Harhar H, El Kamli T, Tabyaoui M. 2020. Chemical composition and antioxidant capacity of *Lepidium sativum* seeds from four regions of Morocco. Evid Based Complement Alternat Med. 2020:7302727. 32714416 10.1155/2020/7302727 PMC7346108

[CIT0024] Darakhshan S, Bidmeshki Pour A, Hosseinzadeh Colagar A, Sisakhtnezhad S. 2015. Thymoquinone and its therapeutic potentials. Pharmacol Res. 95–96:138–158. 10.1016/j.phrs.2015.03.011 25829334

[CIT0025] Dehkordi FR, Kamkhah AF. 2008. Antihypertensive effect of *Nigella sativa* seed extract in patients with mild hypertension. Fundam Clin Pharmacol. 22(4):447–452. 18705755 10.1111/j.1472-8206.2008.00607.x

[CIT0026] Doke S, Guha M. 2014. Garden cress (*Lepidium sativum* L.) seed – an important medicinal source: a review. J Nat Prod Plant Resour. 4:69–80.

[CIT0027] Enayatfard L, Mohebbati R, Niazmand S, Hosseini M, Shafei MN. 2018. The standardized extract of *Nigella sativa* and its major ingredient, thymoquinone, ameliorates angiotensin II-induced hypertension in rats. J Basic Clin Physiol Pharmacol. 30(1):51–58. 30269105 10.1515/jbcpp-2018-0074

[CIT0028] Fasinu PS, Bouic PJ, Rosenkranz B. 2012. An overview of the evidence and mechanisms of herb-drug interactions. Front Pharmacol. 3:69. 22557968 10.3389/fphar.2012.00069 PMC3339338

[CIT0029] Feng M, Deerhake ME, Keating R, Thaisz J, Xu L, Tsaih SW, Smith R, Ishige T, Sugiyama F, Churchill GA, et al. 2009. Genetic analysis of blood pressure in 8 mouse intercross populations. Hypertension. 54(4):802–809. 19652078 10.1161/HYPERTENSIONAHA.109.134569 PMC2854560

[CIT0030] Geberemeskel GA, Debebe YG, Nguse NA. 2019. Antidiabetic effect of fenugreek seed powder solution (*Trigonella foenum-graecum* L.) on hyperlipidemia in diabetic patients. J Diabetes Res. 2019:1–8. 10.1155/2019/8507453 PMC6748210 31583253

[CIT0031] Gholamnezhad Z, Havakhah S, Boskabady MH. 2016. Preclinical and clinical effects of *Nigella sativa* and its constituent, thymoquinone: a review. J Ethnopharmacol. 190:372–386. 27364039 10.1016/j.jep.2016.06.061

[CIT0032] Grassi G. 2018. Metoprolol in the treatment of cardiovascular disease: a critical reappraisal. Curr Med Res Opin. 34(9):1635–1643. 29781321 10.1080/03007995.2018.1479245

[CIT0033] Hamdan A, Haji Idrus R, Mokhtar MH. 2019. Effects of *Nigella sativa* on type-2 diabetes mellitus: a systematic review. Int J Environ Res Public Health. 16(24):4911. 10.3390/ijerph16244911 PMC6950756 31817324

[CIT0034] Ijaz H, Tulain UR, Qureshi J, Danish Z, Musayab S, Akhtar MF, Saleem A, Khan KK, Zaman M, Waheed I, et al. 2017. Review: *Nigella sativa* (prophetic medicine): a review. Pak J Pharm Sci. 30(1):229–234. 28603137

[CIT0035] Jaarin K, Foong WD, Yeoh MH, Kamarul ZY, Qodriyah HM, Azman A, Zuhair JS, Juliana AH, Kamisah Y. 2015. Mechanisms of the antihypertensive effects of *Nigella sativa* oil in L-NAME-induced hypertensive rats. Clinics. 70(11):751–757. 26602523 10.6061/clinics/2015(11)07 PMC4642492

[CIT0036] Johansson T, Weidolf L, Jurva U. 2007. Mimicry of phase I drug metabolism-novel methods for metabolite characterization and synthesis. Rapid Commun Mass Spectrom. 21(14):2323–2331. 17575570 10.1002/rcm.3077

[CIT0037] Kobayashi N, Hara K, Watanabe S, Higashi T, Matsuoka H. 2000. Effect of imidapril on myocardial remodeling in L-NAME-induced hypertensive rats is associated with gene expression of NOS and ACE mRNA. Am J Hypertens. 13(2):199–207. 10701821 10.1016/s0895-7061(99)00117-x

[CIT0038] Kooti W, Hasanzadeh-Noohi Z, Sharafi-Ahvazi N, Asadi-Samani M, Ashtary-Larky D. 2016. Phytochemistry, pharmacology, and therapeutic uses of black seed (*Nigella sativa*). Chin J Nat Med. 14(10):732–745. 28236403 10.1016/S1875-5364(16)30088-7

[CIT0039] Krege JH, Hodgin JB, Hagaman JR, Smithies O. 1995. A noninvasive computerized tail-cuff system for measuring blood pressure in mice. Hypertension. 25(5):1111–1115. 7737724 10.1161/01.hyp.25.5.1111

[CIT0040] Madar Z, Shomer I. 1990. Polysaccharide composition of a gel fraction derived from fenugreek and its effect on starch digestion and bile acid absorption in rats. J Agric Food Chem. 38(7):1535–1539.

[CIT0041] Maghrani M, Zeggwagh NA, Michel JB, Eddouks M. 2005. Antihypertensive effect of *Lepidium sativum* L. in spontaneously hypertensive rats. J Ethnopharmacol. 100(1–2):193–197. 15955648 10.1016/j.jep.2005.02.024

[CIT0042] Majdalawieh AF, Fayyad MW, Nasrallah GK. 2017. Anti-cancer properties and mechanisms of action of thymoquinone, the major active ingredient of *Nigella sativa*. Crit Rev Food Sci Nutr. 57(18):3911–3928. 28140613 10.1080/10408398.2016.1277971

[CIT0043] Mali VR, Mohan V, Bodhankar SL. 2012. Antihypertensive and cardioprotective effects of the Lagenaria siceraria fruit in NG-nitro-L-arginine methyl ester (L-NAME) induced hypertensive rats. Pharm Biol. 50(11):1428–1435. 22994444 10.3109/13880209.2012.684064

[CIT0044] Mansoor GA. 2001. Herbs and alternative therapies in the hypertension clinic. Am J Hypertens. 14(9 Pt 1):971–975. 11587167 10.1016/s0895-7061(01)02172-0

[CIT0045] Mills KT, Bundy JD, Kelly TN, Reed JE, Kearney PM, Reynolds K, Chen J, He J. 2016. Global disparities of hypertension prevalence and control: a systematic analysis of population-based studies from 90 countries. Circulation. 134(6):441–450. 27502908 10.1161/CIRCULATIONAHA.115.018912 PMC4979614

[CIT0046] Mills KT, Stefanescu A, He J. 2020. The global epidemiology of hypertension. Nat Rev Nephrol. 16(4):223–237. 32024986 10.1038/s41581-019-0244-2 PMC7998524

[CIT0047] Modak M, Dixit P, Londhe J, Ghaskadbi S, Devasagayam TP. 2007. Indian herbs and herbal drugs used for the treatment of diabetes. J Clin Biochem Nutr. 40(3):163–173. 18398493 10.3164/jcbn.40.163 PMC2275761

[CIT0048] Mollazadeh H, Afshari AR, Hosseinzadeh H. 2017. Review on the potential therapeutic roles of Nigella sativa in the treatment of patients with cancer: involvement of apoptosis: Black cumin and cancer. J Pharmacopuncture. 20(3):158–172. 30087792 10.3831/KPI.2017.20.019 PMC5633668

[CIT0049] Morani AS, Bodhankar SL, Mohan V, Thakurdesai PA. 2012. Ameliorative effects of standardized extract from *Trigonella foenum-graecum* L. seeds on painful peripheral neuropathy in rats. Asian Pac J Trop Med. 5(5):385–390. 22546656 10.1016/S1995-7645(12)60064-9

[CIT0050] Nagulapalli Venkata KC, Swaroop A, Bagchi D, Bishayee A. 2017. A small plant with big benefits: Fenugreek (*Trigonella foenum-graecum* Linn.) for disease prevention and health promotion. Mol Nutr Food Res. 61:1–26. 10.1002/mnfr.201600950 28266134

[CIT0051] Nair S, Nagar R, Gupta R. 1998. Antioxidant flavonoids in common Indian diet. J Assoc Physicians India. 46:708–710. 11229280

[CIT0052] Palleria C, Di Paolo A, Giofre C, Caglioti C, Leuzzi G, Siniscalchi A, De Sarro G, Gallelli L. 2013. Pharmacokinetic drug-drug interaction and their implication in clinical management. J Res Med Sci. 18(7):601–610. 24516494 PMC3897029

[CIT0053] Patel DK, Prasad SK, Kumar R, Hemalatha S. 2012. An overview on antidiabetic medicinal plants having insulin mimetic property. Asian Pac J Trop Biomed. 2(4):320–330. 23569923 10.1016/S2221-1691(12)60032-X PMC3609288

[CIT0054] Petit PR, Sauvaire YD, Hillaire-Buys DM, Leconte OM, Baissac YG, Ponsin GR, Ribes GR. 1995. Steroid saponins from fenugreek seeds: extraction, purification, and pharmacological investigation on feeding behavior and plasma cholesterol. Steroids. 60(10):674–680. 8539775 10.1016/0039-128x(95)00090-d

[CIT0055] Prajapati VD, Maheriya PM, Jani GK, Patil PD, Patel BN. 2014. *Lepidium sativum* Linn.: a current addition to the family of mucilage and its applications. Int J Biol Macromol. 65:72–80. 24418343 10.1016/j.ijbiomac.2014.01.008

[CIT0056] Ramanathan V, Thekkumalai M. 2014. Role of chrysin on hepatic and renal activities of *N*-nitro-L-arginine-methylester induced hypertensive rats. Int J Nutr Pharmacol Neurol Dis. 4(1):58–63.

[CIT0057] Reddy RR, Srinivasan K. 2011. Dietary fenugreek and onion attenuate cholesterol gallstone formation in lithogenic diet-fed mice. Int J Exp Pathol. 92(5):308–319. 21756271 10.1111/j.1365-2613.2011.00782.x PMC3193144

[CIT0058] Regardh CG, Johnsson G. 1980. Clinical pharmacokinetics of metoprolol. Clin Pharmacokinet. 5(6):557–569. 7002420 10.2165/00003088-198005060-00004

[CIT0059] Rehman NU, Khan AU, Alkharfy KM, Gilani AH. 2012. Pharmacological basis for the medicinal use of *Lepidium sativum* in airways disorders. Evid Based Complement Alternat Med. 2012:596524. 22291849 10.1155/2012/596524 PMC3265128

[CIT0060] Rigby JW, Scott AK, Hawksworth GM, Petrie JC. 1985. A comparison of the pharmacokinetics of atenolol, metoprolol, oxprenolol and propranolol in elderly hypertensive and young healthy subjects. Br J Clin Pharmacol. 20(4):327–331. 2866783 10.1111/j.1365-2125.1985.tb05072.x PMC1400877

[CIT0061] Ripley TL, Saseen JJ. 2014. β-blockers: a review of their pharmacological and physiological diversity in hypertension. Ann Pharmacother. 48(6):723–733. 24687542 10.1177/1060028013519591

[CIT0062] Rizka A, Setiati S, Lydia A, Dewiasty E. 2017. Effect of *Nigella sativa* seed extract for hypertension in elderly: a double-blind, randomized controlled trial. Acta Med Indones. 49(4):307–313. 29348380

[CIT0063] Sung JH, Jo YS, Kim SJ, Ryu JS, Kim MC, Ko HJ, Sim SS. 2013. Effect of lutein on L-NAME-induced hypertensive rats. Korean J Physiol Pharmacol. 17(4):339–345. 23946694 10.4196/kjpp.2013.17.4.339 PMC3741491

[CIT0064] Susalit E, Agus N, Effendi I, Tjandrawinata RR, Nofiarny D, Perrinjaquet-Moccetti T, Verbruggen M. 2011. Olive (*Olea europaea*) leaf extract effective in patients with stage-1 hypertension: comparison with Captopril. Phytomedicine. 18(4):251–258. 21036583 10.1016/j.phymed.2010.08.016

[CIT0065] Syed AA, Lahiri S, Mohan D, Valicherla GR, Gupta AP, Riyazuddin M, Kumar S, Maurya R, Hanif K, Gayen JR. 2016. Evaluation of anti-hypertensive activity of *Ulmus wallichiana* extract and fraction in SHR, DOCA-salt- and L-NAME-induced hypertensive rats. J Ethnopharmacol. 193:555–565. 27720848 10.1016/j.jep.2016.10.008

[CIT0066] Tabassum N, Ahmad F. 2011. Role of natural herbs in the treatment of hypertension. Pharmacogn Rev. 5(9):30–40. 22096316 10.4103/0973-7847.79097 PMC3210006

[CIT0067] Tata CM, Sewani-Rusike CR, Oyedeji OO, Gwebu ET, Mahlakata F, Nkeh-Chungag BN. 2019. Antihypertensive effects of the hydro-ethanol extract of Senecio serratuloides DC in rats. BMC Complement Altern Med. 19(1):52. 30819180 10.1186/s12906-019-2463-2 PMC6394053

[CIT0068] Tavakkoli A, Mahdian V, Razavi BM, Hosseinzadeh H. 2017. Review on clinical trials of black seed (*Nigella sativa ) and Its Active Constituent, Thymoquinone*. J Pharmacopuncture. 20(3):179–193. 30087794 10.3831/KPI.2017.20.021 PMC5633670

[CIT0069] Xiao J, Ke ZP, Shi Y, Zeng Q, Cao Z. 2018. The cardioprotective effect of thymoquinone on ischemia-reperfusion injury in isolated rat heart via regulation of apoptosis and autophagy. J Cell Biochem. 119(9):7212–7217. 29932232 10.1002/jcb.26878

[CIT0070] Yadav UC, Baquer NZ. 2014. Pharmacological effects of *Trigonella foenum-graecum* L. in health and disease. Pharm Biol. 52(2):243–254. 24102093 10.3109/13880209.2013.826247

[CIT0071] Yoshikawa M, Murakami T, Komatsu H, Murakami N, Yamahara J, Matsuda H. 1997. Medicinal foodstuffs. IV. Fenugreek seed. (1): structures of trigoneosides Ia, Ib, IIa, IIb, IIIa, and IIIb, new furostanol saponins from the seeds of Indian *Trigonella foenum-graecum* L. Chem Pharm Bull. 45(1):81–87. 10.1248/cpb.45.81 9023970

[CIT0072] Zhao X, Ho D, Gao S, Hong C, Vatner DE, Vatner SF. 2011. Arterial pressure monitoring in mice. Curr Protoc Mouse Biol. 1:105–122. 21686061 10.1002/9780470942390.mo100149 PMC3115725

